# Clinical impairments associated with ankle disability in patients with acute lateral ankle sprain

**DOI:** 10.3389/fpubh.2025.1617269

**Published:** 2025-07-02

**Authors:** Young-Hyun Lee, Kang-Jun Lee, Seung-Hee Nam, Kyung-Min Kim

**Affiliations:** Department of Sport Science, Sungkyunkwan University, Suwon-si, Republic of Korea

**Keywords:** patient-oriented outcomes, self-reported measures, disablement, ankle dysfunction, acute injury

## Abstract

**Background:**

Acute lateral ankle sprains (ALAS) are common musculoskeletal injuries among physically active individuals. While various impairments occur following ALAS, limited information is available on the factors contributing to ankle disability. This study aimed to investigate the association between the clinical impairments and ankle disability in patients with ALAS.

**Methods:**

We conducted a cross-sectional design with 55 ALAS patients within two weeks of injury. Clinical impairments, including inflammatory symptoms (e.g., pain and swelling), restricted total ankle motion, joint laxity (e.g., anterior drawer test; ADT, inversion talar tilt test; ITT), and functional limitation were analyzed for their association with ankle disability assessed by the Foot and Ankle Ability Measure (FAAM) in Activities of Daily Living (ADL) and Sports (S) subscales.

**Results:**

Multiple regression analysis revealed significant models that explained approximately 70% of the variance in FAAM subscales. The results indicated that swelling (*β* = −0.620 for FAAM-ADL, *p* < 0.01, *β* = −0.765 for FAAM-S, *p* < 0.01) and pain (*β* = −0.347 for FAAM-ADL, *p* < 0.01, *β* = −0.470 for FAAM-S, *p* < 0.01) were the most significant contributors to ankle disability in both subscales. Joint laxity measured by the ITT (*β* = −0.199, *p* < 0.05) negatively affected sports-related disability while restricted total ankle motion (*β* = 0.307, *p* < 0.05) had a positive effect. However, functional limitation was not significant in both subscales.

**Conclusion:**

These findings highlight the clinical impairments associated with ALAS, which contribute to ankle disability. Our results suggest that these impairments may be relevant considerations when designing early rehabilitation strategies (e.g., swelling reduction, pain control, and joint stability) for individuals with ALAS.

## 1 Introduction

Ankle sprains are among the most prevalent musculoskeletal injuries incurred in the physically active population (1). The incidence rate of acute ankle sprains ranges from 2 to 7 cases per 1,000 person-years, with approximately 2 million occurring annually in the United States (2). In addition to their high occurrence in the general population, ankle sprains are also prevalent in athletes, accounting for about 15% of all athletic injuries and an incidence rate of 0.93 per 1,000 athletic exposures (3, 4). Acute lateral ankle sprains (ALAS) are the most common type of ankle sprains in the population. Many patients with ALAS, up to 72%, suffer from residual symptoms such as pain and swelling, muscle weakness, and joint instability (5). Furthermore, patients with ALAS are more prone to sustaining recurrent ankle injuries, which may limit physical activities and contribute to the development of ankle disability, thereby affecting their health-related quality of life (6). Approximately one-third of patients with ALAS develop chronic ankle instability (CAI), a clinical condition that not only involves joint instability but also indicates a chronic pathological state (7). This condition has been associated with an early onset of more debilitating joint disorders, such as osteoarthritis (8–10). Despite these potential consequences of ALAS, patients often do not seek medical treatment due to the public perception of ALAS as a minor injury (11). Therefore, it is crucial to recognize ALAS as an injury that can significantly impact one’s quality of life and requires professional treatment.

Various methods for treating patients with ALAS have been introduced in the literature, but they appear to be suboptimal (12). This may be due to the current rehabilitation paradigm, which tends to focus more on pathological conditions with less attention to patient perspectives into rehabilitation (13). Patient-reported outcomes (PROs) are a key measure of patient-oriented outcomes, used to gather information directly from patients regarding their health condition and the effects of treatment (14). PROs provide direct insight into how patients perceive their recovery and the impact of their injury on daily and sports life, allowing for adjustments in treatment plans to better meet their needs (15). The Foot and Ankle Ability Measure (FAAM) is a PRO tool specifically developed to assess self-reported ankle function in individuals with foot and ankle disorders (6, 16). It is widely used to evaluate ankle disability following ankle sprains, with substantial evidence indicating its relevance in patients with CAI (7, 17–21). To better understand and address ankle disability in rehabilitation, previous research has identified clinical impairments, defined as observable and measurable physical deficits such as pain, swelling, joint laxity, reduced range of motion, and functional limitations that interfere with normal ankle function, as contributing factors (9, 22, 23). In particular, impairments including restricted ankle motion (24), mechanical laxity (25–27), and functional limitation (28), negatively affect perceived disability, suggesting that persistent issues associated with CAI such as restricted motion, joint laxity, and functional limitation contribute to ankle disability. These findings underscore the need for a comprehensive rehabilitation approach that not only addresses clinical impairments but also incorporates patient perspectives and values to achieve better outcomes (7).

In contrast to the growing evidence of CAI research on ankle disability, studies investigating clinical impairments associated with ankle disability in patients with ALAS remain limited. Moreover, existing research has yielded inconsistent findings. While some studies report significant negative associations between impairments and patient-reported functionality, others find no meaningful impact (29–32). These inconsistencies may stem from a predominant focus on isolated impairments, failing to account for the relationship of multiple concurrent impairments often present after ALAS. Addressing this gap requires a more comprehensive approach that considers the combined and individual contributions of multiple clinical impairments to ankle disability. Therefore, the purpose of the current study was to identify the common clinical impairments following ALAS and determine their association with ankle disability, as measured by FAAM scores. Although the FAAM has been primarily validated in individuals with CAI, recent studies have demonstrated its applicability in ALAS populations (33–35). Moreover, current clinical practice guidelines recommend the FAAM for monitoring patient-reported disability in both acute and chronic ankle conditions (36), supporting its relevance and appropriateness for use in this study.

## 2 Materials and methods

The current study is a descriptive, cross-sectional study examining common signs and symptoms, known as clinical impairments, associated with ankle disability in patients with ALAS. Ankle disability served as the dependent variable in the study, with common impairments acting as independent variables. These impairments included inflammatory symptoms (ankle pain and swelling), restricted total ankle motion, ankle joint laxity, and functional limitation. These variables were classified as clinical impairments based on their alignment with the body function and structure domain of the International Classification of Functioning, Disability and Health (ICF) framework (36), and their consistent identification in prior ankle sprain literature (9, 22, 23) as measurable deficits commonly observed in individuals with ALAS. We designated these variables as conceptual contributors due to the cross-sectional design.

### 2.1 Participants

*A priori* sample size estimation was conducted using G*Power (v3.1.9.2) for multiple regression analysis with six predictors: (1) current pain, (2) swelling, (3) restricted total ankle motion, (4) ankle joint laxity in the anteroposterior direction, (5) ankle joint laxity in the mediolateral direction, and (6) functional limitation. A pilot study involving 15 ALAS patients yielded an R^2^ of 0.26 (*f*^2^ = 0.35). Based on this large effect size, with *α* = 0.05 and desired power = 0.80, the minimum required sample was estimated to be forty-three (*N* = 43). To account for potential attrition and ensure sufficient power, we aimed to recruit more than this minimum and successfully enrolled 55 participants. They were recruited from a university community through emails, flyers, phone calls, and referrals from the university hospital or local clinics. Eligible participants had experienced ALAS within two weeks prior to enrollment and exhibited clear clinical symptoms, including pain or tenderness, swelling, discomfort, and functional loss at the time of testing (9). A certified athletic trainer performed standardized ankle evaluations, following protocols consistent with previous studies (33–35), to confirm the diagnosis of ALAS and rule out other types of ligament sprains. The evaluation included assessments of the injury mechanism, application of the Ottawa Ankle/Foot Rule to exclude fractures, presence of ecchymosis, tenderness at specific ankle points, active range of motion, joint laxity tests, functional ability ratings, and injury classification (Grade I, II, or III). Participants were further screened to ensure they had no history of neurological injuries or surgeries, seizure disorders, balance or vestibular dysfunctions, recent low back pain, or self-reported pregnancy. To minimize the potential influence of recent interventions on symptom presentation, all participants were instructed to discontinue any symptom management or treatment activities (e.g., medication, compression, or therapeutic modalities) at least six hours prior to participation in the study. This precautionary measure is consistent with previous research protocols in acute ankle sprain studies (33, 34). The study received approval from the university’s institutional review board, and all participants provided written informed consent before participation.

### 2.2 Experimental procedures

#### 2.2.1 Inflammatory symptoms

Pain levels in this study were measured using the visual analog scale (VAS), a 10 cm horizontal line marked from ‘0’ (indicating no pain) to ‘10’ (indicating the worst pain imaginable) (37). Patients indicated their perceived pain intensity by marking a point on the line, with higher score reflecting greater pain intensity (37). The VAS is a reliable tool for measuring acute pain, with an intraclass correlation (ICC) score of 0.97 (38). Ankle swelling was evaluated using the figure-of-eight method, which involves comparing the circumference of the injured ankle with that of the uninjured ankle and recording the difference in centimeters. The figure-of-eight method has been reported as a valid and clinically applicable technique for quantifying ankle joint swelling, with almost perfect consistency (i.e., intraclass and interrater correlation coefficients of 0.99) (39). The procedure involves starting the tape at the groove between the lateral malleolus and tibialis anterior tendon. The tape is wrapped medially over the navicular tubercle, under the foot across the base of the 5^th^ metatarsal, dorsally toward the medial malleolus, around the Achilles tendon, and back to the starting point. The average of three measurements was used for data analysis, with higher number indicating the greater swelling (39).

#### 2.2.2 Restricted total ankle motion

Restricted total ankle motion was qualitatively assessed by a certified athletic trainer using a structured grading system. This assessment relied on visual observation, making it inherently subjective. However, similar grading methods have been adopted in prior ALAS studies to evaluate ankle mobility (33–35) and they remain widely used in clinical settings due to their feasibility, time efficiency, and minimal equipment requirements. The patient’s active range of motion (ROM) during dorsiflexion, plantarflexion, inversion, and eversion was observed and compared to the contralateral, uninjured ankle. ROM was evaluated in a non-weight bearing, sitting position with the knee flexed at 90°. Performance was rated on a 4-point scale: (1) no restriction, (2) mild restriction, (3) moderate restriction, (4) severe restriction. The scores ranged from 0 to 3, with higher scores indicating greater restriction of motion (40).

#### 2.2.3 Ankle joint laxity

Ankle joint laxity was assessed using the anterior drawer test (ADT) and inversion talar tilt test (ITT) as they are commonly performed in the clinical settings (41). Both tests are reliable for measuring ankle joint laxity as the intra-rater reliability was 0.74 for the ITT (good) and 0.65 for the ADT (moderate) (42). For the ADT, the patient sat with the knee flexed at 90°, and the examiner grasped the heel and area above the malleolus, pulling the heel forward (43). For the ITT, the patient’s foot was unsupported in 10–20° of plantarflexion, and the examiner inverted the hindfoot while palpating the talus to detect tilting (44). Laxity was assessed by comparing results to the uninjured side (41). These test outcomes are graded on a scale of 1 to 5: (1) very hypomobile; (2) slightly to moderately hypomobile, (3) normal, (4) slightly to moderately hypermobile, (5) very hypermobile, where higher scores represent a greater degree of joint laxity (33, 34).

#### 2.2.4 Functional limitation

Functional limitation following ALAS was assessed using a series of weight-bearing and functional performance tests. These tests may be subjective, as they rely on assessments by certified athletic trainer. However, they have been used in previous ALAS studies to assess functional limitation (33–35). There were 6 tasks/tests, including (1) incapable of bearing weight (reliant on crutches for ambulation), (2) capable of bearing partial weight (walk-through crutch gait or cane assistance), (3) capable of bearing full weight without assistance, but some degree of gait asymmetry apparent, (4) normal walking gait, (5) capable of unilateral vertical hopping on involved extremity without pain or apprehension, and (6) capable of unilateral horizontal hopping on involved extremity without pain or apprehension. The initial task level was chosen according to each participant’s observed weight-bearing status at the time of assessment. Participants then attempted tasks in ascending order, advancing only if they completed the previous level without pain, compensatory movement, or apprehension. If a participant could not perform a given task, their final score was recorded as the highest level successfully completed. Higher scores (range: 1–6) indicate better weight-bearing capacity and ankle function (33).

#### 2.2.5 Foot and ankle ability measure (FAAM)

The foot and ankle ability measure (FAAM) questionnaire was commonly used to assess the functionality of the foot and ankle (21, 34, 45, 46). The test–retest reliability of the FAAM was 0.89 for the Activities of Daily Living (ADL) subscale and 0.87 for the Sports (S) subscale. Both subscales have demonstrated responsiveness to changes in functional status (16) in individuals with foot and/or ankle disorders such as ALAS. The FAAM-ADL questionnaire comprises 21 questions assessing ankle disability in activities such as standing, squatting, and walking to evaluate the patient’s ability to perform everyday tasks. On the other hand, the FAAM-S includes 8 questions focused on dynamic activities like rapid starts and stops, jumping, landing, and cutting movements to measure the impact of the ankle condition on the patient’s ability to participate in sports and other physically demanding activities (16). Responses to each question are recorded on a 5-point Likert scale, reflecting the perceived difficulty of the respective activity: 0 for “unable to do,” 1 for “extreme difficulty,” 2 for “moderate difficulty,” 3 for “slight difficulty,” and 4 for “no difficulty at all.” The score range for FAAM-ADL is 0 to 84, while the FAAM-S ranges from 0 to 32. Each subscale’s score is converted into a percentage (16). A percentage score below 90% on the FAAM-ADL, or below 80% on the FAAM-S, indicates disabled ankle, with lower scores in both subscales denoting more severe dysfunction (47).

### 2.3 Statistical analysis

Descriptive analyses were conducted for all variables. Continuous variables were summarized as means with standard deviations (SDs) or medians with ranges, while categorical variables were presented as frequencies and percentages. Spearman’s correlation coefficient was used to assess relationships between variables. A multiple regression analysis was conducted to identify the clinical impairments that are associated with ankle disability in patients with ALAS. Before the analysis, all continuous variables were checked for normality, categorical variables were converted to numerical codes according to their ordinal nature (48). The regression model evaluated the relationship between each FAAM scores and associated factors, including current pain, ankle swelling, restricted total ankle motion, ankle joint laxity observed during the ADT and ITT, and functional limitation. Multicollinearity among predictors was assessed using the Variance Inflation Factor (VIF), with a threshold value of VIF > 10 indicating high multicollinearity (49, 50). Separate regression models were constructed for FAAM-ADL and FAAM-S scores as dependent variables. The alpha level for determining the significance of the coefficients was set at 0.05. The regression coefficients (R-squared and adjusted R-squared values) were interpreted as follows: < 0.3 (negligible), 0.3–0.5 (low/weak), 0.5–0.7 (moderate), 0.7–0.9 (high/strong), and 0.9–1.0 (very high/very strong) (51). Statistical analyses were performed using SPSS 29.0 statistical software (SPSS, Chicago, IL, USA).

## 3 Results

The study included 55 participants with ALAS, comprising 28 males (50.9%) and 27 females (49.1%), with a mean age of 21 years. The median time since injury was 2 days (range: 1–11), and the median number of previous ankle sprains was 1 (range: 0–9). Most participants had Grade I injuries (74.5%), while 25.5% had Grade II injuries as shown in Table 1.

**Table 1:** Participant demographics.

Variables	Descriptive statistics
Sex	Males (*n* = 28, 50.9%) Females (*n* = 27, 49.1%)
Age (yrs.)	21 ± 3.55
Height (cm)	173.5 ± 8.38
Weight (kg)	71.3 ± 11.41
Time since injury (day)^a^	2 (1–11)
Number of previous ankle sprains^a^	1 (0–9)
Injury grade	Grade I (*n* = 41, 74.5%) Grade II (*n* = 14, 25.5%)

^a^The data are presented as medians and ranges.

Table 2 presents the description of clinical impairments. The mean ankle swelling was 1.5 cm (0.7–3.1 cm), and the median VAS pain score was 3 (0.3–5.9). Restricted total ankle motion was mostly mildly (41.8%) or moderately restricted (27.3%), with only 9.1% showing normal motion. Slight hypermobility was common in both the anterior drawer test (63.6%) and talar tilt test (47.3%), while normal laxity was observed in 27.3 and 38.2%, respectively. Regarding functional limitation, 23.6% had partial weight-bearing, while 70.9% exhibited full weight-bearing with gait asymmetry. Only 5.5% demonstrated a normal gait.

**Table 2:** Descriptive summary of clinical impairments.

Variables	Descriptive statistics
Ankle swelling (cm)^a^	1.5 (0.7–3.1)
Current pain (cm)^a^	3 (0.3–5.9)
Restricted total ankle motion	Severely restricted (*n* = 5, 7.3%) Moderately restricted (*n* = 15, 27.3%) Mildly restricted (*n* = 23, 41.8%) Normal (*n* = 4, 9.1%)
Ankle joint by anterior drawer test	Slightly hypomobile (*n* = 1, 1.8%) Normal (*n* = 15, 27.3%) Slightly hypermobile (*n* = 35, 63.6%) Very hypermobile (*n* = 4, 7.3%)
Ankle joint by inversion talar tilt test	Slightly hypomobile (*n* = 3, 5.5%) Normal (*n* = 21, 38.2%) Slightly hypermobile (*n* = 26, 47.3%) Very hypermobile (*n* = 5, 9.1%)
Functional limitation	Partial weight-bearing (*n* = 13, 23.6%) Full weight-bearing with some degree of gait asymmetry (*n* = 39, 70.9%) Normal walking gait (*n* = 3, 5.5%)

^a^The data are presented as medians and ranges.

Before model fitting, we evaluated multicollinearity among the six predictors. Spearman correlations revealed several moderate associations (Table 3), with the strongest observed between current pain and restricted total ankle motion (*ρ* = 0.72) and between swelling and restricted total ankle motion (*ρ* = 0.69). We next calculated variance inflation factors (VIFs) and found that values ranged from 1.12 to 3.33 (Table 3), well below the conservative cutoff of 10 that indicates severe multicollinearity. These findings demonstrate only mild shared variance among predictors; consequently, no further statistical adjustment was required at this stage, and the set of variables was deemed suitable for subsequent regression modeling.

**Table 3:** Correlation matrix and multicollinearity diagnostics of clinical impairments associated with ankle disability in ALAS patients.

Variables	Swelling	Current pain	Restricted total ankle motion	Ankle joint laxity_ADT	Ankle joint laxity_ITT	Functional limitation	VIF
Swelling	-	0.48	0.69	−0.16	−0.02	−0.48	2.14
Current pain	0.48	-	0.72	−0.02	−0.08	−0.59	2.56
Restricted total ankle motion	0.69	0.72	-	−0.12	−0.07	−0.54	1.81
Ankle joint laxity_ADT	−0.16	−0.02	−0.12	-	0.31	−0.03	3.33
Ankle joint laxity_ITT	−0.02	−0.08	−0.07	0.31	-	0.01	1.17
Functional limitation	−0.48	−0.59	−0.54	−0.03	0.01	-	1.12

ADT = anterior drawer test; ITT = inversion talar tilt test; VIF = variance inflation factor.

The multiple regression analysis of FAAM-ADL scores revealed a significant model (F_6,48_ = 18.74, *p* < 0.001) with an R^2^ of 0.701, indicating that approximately 70.1% of the variability in FAAM-ADL scores is explained by the contributors. The model’s adjusted R^2^ was 0.664, reflecting a strong fit given the number of contributors used. Significant contributors included current pain [*B* = −4.24, 95% CI (−7.35, −1.13), *p* = 0.01], and ankle swelling [*B* = −14.08, 95% CI (−19.36, −8.80), *p* < 0.001]. The significant contributors in the FAAM-ADL model highlight the importance of managing current pain and ankle swelling to improve ankle disability perceived during daily activities of living. Specifically, each unit increase in current pain results in a 4.24-point decrease in FAAM-ADL scores. Similarly, ankle swelling has a substantial negative effect, with each unit increase leading to a 14.08-point decrease in scores. The confidence intervals for these contributors did not cross zero, indicating that significant relationships exist as shown in Table 4. However, laxity assessed by ADT and ITT, restricted total ankle motion, and functional limitation, were not statistically significant.

**Table 4:** Clinical impairments associated with ankle disability perceived during activity of daily living in ALAS patients.

Variables	*B*	SE	*t*	95% Confidence interval		Beta (*β*)
				Lower	Upper	
Constant	95.39	18.38	5.19	58.43	132.35	
Swelling	−14.08	2.63	−5.36*	−19.36	−8.8	−0.62
Current pain	−4.24	1.55	−2.74*	−7.35	−1.13	−0.35
Restricted total ankle motion	−1.1	3.39	−0.32	−5.72	7.91	0.05
Ankle joint laxity_ADT	0.84	2.54	0.33	−4.26	5.94	0.03
Ankle joint laxity_ITT	−2.14	2.05	−1.04	−6.27	1.99	−0.09
Functional limitation	1.36	3.73	0.36	−6.15	8.87	0.04

*Indicates significant contributors (*p* < 0.05).

*B*, unstandardized beta; SE, standard error; Beta, standardized beta; ADT, anterior drawer test; ITT, inversion talar tilt test.

Additionally, the multiple regression analysis of FAAM-S scores yielded a significant model (F_6,48_ = 18.54, *p* < 0.001) with an R^2^ of 0.696, indicating that approximately 69.6% of the variability in FAAM-S scores is explained by the associated factors, with an adjusted R^2^ of 0.658. Significant factors included ankle swelling [*B* = -17.63, 95% CI (−23.01, −12.25), *p* < 0.001], current pain [*B* = −5.84, 95% CI (−9.00, −2.67), *p* < 0.001], restricted total ankle motion [*B* = 7.33, 95% CI (0.39, 14.28), *p* = 0.04], and laxity assessed by ITT [*B* = −5.14, 95% CI (−9.35, −0.93), *p* = 0.02] showed meaningful associations with FAAM-S scores, as presented in Table 5. However, laxity assessed by ADT and functional limitation were not statistically significant. Specifically, each unit increase in current pain results in a 5.84-point decrease in FAAM-S scores. Similarly, ankle swelling has a major negative effect, with each unit increase associated with a 17.65-point decrease in scores. Additionally, increased ankle joint laxity measured by ITT correlated with a 4.95-point decrease per unit. Conversely, restricted total ankle motion demonstrates a positive relationship, where each unit increase corresponds to a 7.33-point increase in FAAM-S scores. The confidence intervals for these contributors did not cross zero, confirming the presence of these relationships.

**Table 5:** Clinical impairments associated with ankle disability perceived during sports in ALAS patients.

Variables	*B*	SE	*t*	95% Confidence interval		Beta (*β*)
				Lower	Upper	
Constant	85.3	18.73	4.55	47.64	122.96	
Swelling	−17.63	2.68	−6.59*	−23.01	−12.25	−0.76
Current pain	−5.84	1.57	−3.71*	−9.0	−2.67	−0.47
Restricted total ankle motion	7.33	3.45	2.12*	0.39	14.28	0.31
Ankle joint laxity_ADT	3.19	2.58	1.23	−2.0	8.39	0.11
Ankle joint laxity_ITT	−5.14	2.09	−2.46*	−9.35	−0.93	−0.21
Functional limitation	−2.38	3.8	−0.62	−10.03	5.27	−0.07

^*^Indicates significant contributors (*p* < 0.05).

*B*, unstandardized beta; SE, standard error; Beta, standardized beta; ADT, anterior drawer test; ITT, inversion talar tilt test.

## 4 Discussion

We found that ankle swelling and current pain emerged as the most significant contributors, with substantial negative impacts on FAAM scores. Specifically, the FAAM-ADL model demonstrated that approximately 70.1% of the variability in scores was explained by the contributors, with ankle swelling having the strongest effect, followed by current pain. Similarly, the FAAM-S model explained 69.6% of the variability, also identifying ankle swelling and current pain as major determinants, alongside joint laxity (ITT) and restricted total ankle motion. Importantly, unlike previous studies (29–32) that primarily focused on pair-wise relationships between individual factors and ankle functionality, the current study utilized multiple regression analysis to investigate multiple factors simultaneously within a single model. By accounting for multiple variables at once, the present study provides a more robust and nuanced understanding of the relative impact of common clinical impairments in patients with ALAS. This approach offers a clearer picture of the multifaceted nature of ankle disability and how it may be addressed in both clinical and athletic contexts.

### 4.1 Inflammatory symptoms

We revealed that swelling emerged as the most significant factor contributing to ankle disability, followed by pain across both FAAM scales. These results highlight that swelling and pain are key contributors to ankle disability, aligning with the general understanding that inflammatory symptoms negatively impact ankle functionality. However, there are few studies examining the relationship of either ankle pain or swelling with ankle disability in patients with ALAS. While two studies of ALAS patients reported these clinical impairments negatively affect ankle disability (30, 32), another study did not support the relationship (31). For instance, Khazaei et al. (32) reported a significant correlation between pain and functional limitation in patients with ALAS (*t* = 2.16, *p* = 0.04), supporting our result that pain is a critical factor influencing ankle disability. Similarly, Man et al. (30) observed a slight negative trend (*r* = −0.003) between swelling and ankle function, suggesting a potential association between increased swelling and reduced functionality, although it did not reach statistical significance. In contrast, Pugia et al. (31) found no significant relationship between swelling and ankle functionality. This discrepancy may be due to multiple factors such as small sample size. Further studies are warranted to draw the clear conclusion about the relationship. Nonetheless, the current study with larger sample size provides unique evidence of the concurrent effects of ankle swelling and pain, providing a more comprehensive understanding of their relationship and impact on ankle disability. This approach goes beyond the isolated analyses seen in prior studies, which may have overlooked critical interactions among common clinical impairments. Therefore, the observed significance of swelling and pain as contributors to ankle disability underscores the importance of targeted interventions addressing these symptoms in post-ALAS rehabilitation.

### 4.2 Restricted total ankle motion

This study revealed a positive association between restricted total ankle motion and ankle disability, which contrasts with our initial hypothesis. Previous literature generally suggests that limited ankle motion following ALAS negatively affects an athlete’s ability to train and compete, hindering physical activity and functional recovery (52). As a result, previous research suggested early restoration of ROM or functional treatment are often recommended to expedite recovery and restore functional capacity (53–55). However, early mobilization or functional treatment during rehabilitation may present disadvantages (56, 57). Some studies have indicated that early mobilization can increase pain and instability shortly after treatment (56) and a one-year follow-up study reported that individuals who underwent early mobilization experienced slightly more residual subjective complaints compared to those who received cast immobilization (57). From this perspective, one could speculate that a greater degree of motion restriction might be advantageous during the early phase of rehabilitation. Nonetheless, this interpretation must be viewed with caution because several methodological and clinical factors could have inflated the observed relationship. First, restricted total ankle motion was evaluated using a 4-point visual grading scale, an inherently subjective tool that, although widely employed for its speed and practicality, has not yet demonstrated formal reliability within our cohort. Second, ankle-motion scores may have been confounded by acute symptoms such as pain and swelling, which can reduce a patient’s willingness to move to end-range and thus bias the visual assessment toward restricted. Third, psychological factors, particularly kinesiophobia or fear of re-injury, can lead patients to self-limit motion and simultaneously report higher disability, further complicating the association between measured restriction and FAAM scores. Taken together, these considerations suggest that future studies should employ objective, reliability-tested motion measures and longitudinal designs to confirm whether early motion restriction truly mitigates or merely masks ankle disability after ALAS.

### 4.3 Ankle joint laxity

Ankle joint laxity was significantly negatively associated with sports-related ankle disability in our study. Previous researchers have largely prioritized the association between the CAI and ankle disability because laxity often persists beyond the initial injury period, resulting in a prolonged disability (58). For instance, Hubbard (59) found a moderate negative correlation between mechanical joint laxity, such as anterior displacement (*r* = −0.65, *p* = 0.013 for FADI, *r* = −0.88, *p* < 0.001 for FADI-Sports) and inversion rotation (*r* = −0.53, *p* = 0.013 for FADI, *r* = −0.45, *p* = 0.013 for FADI-Sports), and ankle disability in CAI; Lee et al. (60) reported that inversion/eversion displacements were negatively correlated with self-reported function (*r* = −0.33, *p* < 0.001 for FAAM-ADL, *r* = −0.35, *p* < 0.001 for FAAM-S). These findings highlight that excessive laxity not only contributes to immediate instability but also has a detrimental impact on long-term recovery and performance. In our study, laxity evaluated using ITT had a significantly negative impact on ankle disability, unlike ADT, indicating that increased inversion/eversion laxity can significantly contribute to ankle disability in patients with ALAS. Given that inversion rotation often increases after ALAS, ITT is more intuitive to capture functional limitation related to talocrural and subtalar instability (58, 61). These findings underscore the importance of addressing joint laxity early in the recovery process, as excessive inversion/eversion displacement can negatively impact ankle function. Rehabilitation programs that prioritize joint stability, particularly by enhancing medial displacement control, may help mitigate disability after ALAS.

### 4.4 Functional limitation

The role of functional limitation, assessed by a series of weight-bearing and performance tests, in ankle disability following ALAS was explored in this study, but they were not found to be statistically significant contributors to the FAAM-ADL and FAAM-S scores. Although previous research has demonstrated that weight bearing status was negatively associated with the ankle disability (rho = 0.68, *p* < 0.001), indicating the functional limitation is positively related to ankle disability (31), our findings suggest that this relationship may not be straightforward. One possible explanation for the lack of a significant association between functional limitation and ankle disability is that weight-bearing ability can vary significantly among individuals, regardless of the injury’s severity. In our study, most patients could perform at least partial weight-bearing to normal gait, which may have reduced the variability in weight-bearing scores. Differences in subjects’ physical attribute, their reaction to injury, pain tolerance could influence how they perceive and perform (29, 31). As a result, the association with FAAM scores may not have reached statistical significance.

Despite the lack of statistical significance, it is well documented that functional limitation is prevalent in the early stages after an ankle sprain and can persist long-term in some individuals (62). This limitation is crucial considerations, particularly as they are included in the return-to-play criteria supported by an international multidisciplinary consensus, highlighting their importance in clinical decision-making and rehabilitation protocols (63). Therefore, although our findings did not reveal a significant correlation, the importance of monitoring and addressing functional limitation in clinical practice remains crucial, particularly for optimizing recovery and ensuring safe return to activity.

### 4.5 Clinical implications

Our findings illustrate a clear connection between active pathology, impairment, and disability within the disablement model, providing evidence for prioritizing specific factors in rehabilitation strategies for ALAS. Given that pain and swelling are the most significant contributors to ankle disability, clinicians should focus on managing these symptoms during the acute phase. Evidence-based interventions such as non-steroidal anti-inflammatory drugs (NSAIDs), cryotherapy, and compression may effectively reduce swelling and pain, potentially mitigating disability and enhancing recovery (64, 65). While restricted total ankle motion was positively associated with sports-related disability, some degree of early immobilization could be protective by controlling inflammation and reducing acute symptoms (66). Clinicians should balance the need for early immobilization with the importance of mobilization to prevent long-term functional deficits, with adjustments based on the patient’s response and recovery stage. The observed association between joint laxity and ankle disability suggests that ankle instability may warrant targeted attention during rehabilitation; however, interventional research is needed to confirm the benefit of stability-focused interventions. More broadly, addressing the impairments identified in this study during the acute phase may help optimize recovery, although causality cannot be inferred from our cross-sectional data. Finally, incorporating patient-reported outcomes into clinical decision-making may support a more patient-centered approach and should be examined in future trials.

### 4.6 Limitations and recommendations for future study

Our study had some limitations. First, the cross-sectional design used in this study limits our ability to determine causality between these clinical impairments and ankle disability. Cross-sectional studies cannot track the progression of these impairments or their long-term impacts on ankle function. Therefore, prospective longitudinal studies are necessary to observe how these impairments evolve and to clarify the direction and strength of their relationship with ankle disability over time. Second, clinical impairments commonly observed following ALAS are not limited to those examined in the present study. For instance, somatosensory deficits, such as altered joint position sense, reduced joint kinesthesia, and impaired tactile sensitivity, have been reported after ALAS and may significantly influence self-reported ankle disability (36, 67). Future studies should incorporate a more comprehensive assessment of clinical measures, including somatosensory function, to better elucidate the multifactorial nature of ankle disability during the acute phase of ALAS. In addition, we did not collect detailed information regarding any treatment or symptom management (e.g., medication, compression, or therapeutic exercises) participants may have received between injury and testing. Although participants were instructed to refrain from such interventions for at least six hours prior to assessment to minimize potential effects, the absence of formal documentation remains a limitation and may have influenced the clinical presentation of pain and swelling. Another limitation is that participants were recruited from a university setting, which may limit the generalizability of the findings to broader age groups and activity levels. Future study should aim to include a more diverse population to improve external validity and examine how these factors influence clinical impairments and ankle disability. Lastly, a systematic review (68) has highlighted the importance of assessing psychological factors, such as injury-related fear or kinesiophobia, which can significantly impact ankle function and rehabilitation outcomes after ALAS. Although this study did not include these psychological factors, future research on ALAS should consider incorporating psychological assessments to provide a more comprehensive understanding of the factors that influence recovery.

## 5 Conclusion

This study identified inflammatory symptoms, restricted total ankle motion, and ankle joint laxity as significant contributors to ankle disability following ALAS, whereas functional limitation did not show a significant association. By utilizing the multiple regression analysis, we highlighted the multifaceted relationship between these clinical impairments. These findings may help inform rehabilitation strategies by highlighting the potential relevance of managing inflammatory symptoms such as swelling reduction, pain control, joint stability. However, additional interventional studies are needed to determine whether specifically addressing these impairments leads to meaningful improvements in functional recovery and patient-reported outcomes.

## Data Availability

The raw data supporting the conclusions of this article will be made available by the authors, without undue reservation.
