# Correction: Clinical impairments associated with ankle disability in patients with acute lateral ankle sprain

**DOI:** 10.3389/fpubh.2025.1699965

**Published:** 2025-09-17

**Authors:** Frontiers Production Office

**Affiliations:** Frontiers Media SA, Lausanne, Switzerland

**Keywords:** patient-oriented outcomes, self-reported measures, disablement, ankle dysfunction, acute injury

There was a mistake in Table 3 as published. During proofing, a revised Table was submitted which correctly showed the value of VIF for Functional limitation as “1.12” but this value erroneously appeared as a dash “(–)” instead. The corrected Table 3 appears below.

**Table 3:** Correlation matrix and multicollinearity diagnostics of clinical impairments associated with ankle disability in ALAS patients.

**Variables**	**Swelling**	**Current pain**	**Restricted total ankle motion**	**Ankle joint laxity_ADT**	**Ankle joint laxity_ITT**	**Functional limitation**	**VIF**
Swelling	–	0.48	0.69	−0.16	−0.02	−0.48	2.14
Current pain	0.48	–	0.72	−0.02	−0.08	−0.59	2.56
Restricted total ankle motion	0.69	0.72	–	−0.12	−0.07	−0.54	1.81
Ankle joint laxity_ADT	−0.16	−0.02	−0.12	–	0.31	−0.03	3.33
Ankle joint laxity_ITT	−0.02	−0.08	−0.07	0.31	–	0.01	1.17
Functional limitation	−0.48	−0.59	−0.54	−0.03	0.01	–	1.12

ADT, anterior drawer test; ITT, inversion talar tilt test; VIF, variance inflation factor.

The original version of this article has been updated.

